# Supplementary material for: Serologic and Molecular Diagnosis of Anaplasma platys and Ehrlichia canis Infection in Dogs in an Endemic Region
Source: Pathogens. 2020 Jun 19;9(6):488. doi: 10.3390/pathogens9060488 (PMC7350331; doi:10.3390/pathogens9060488)
Supplement: Supplementary file 1 [file pathogens-09-00488-s001.pdf]

Supplemental File-1

This supplemental file presents tables (Tables S1-S15) showing comparison of association of various relevant clinical pathology parameters from a subset of dogs with *A. platys* and *E. canis* POC and PCR test results and Table S16 showing the reference values used in this study. Contingency tables were prepared and analyzed using Fisher's exact test in GraphPad QuickCalcs software. *A. platys* and *E. canis* POC and PCR test results were compared with each of the clinical pathology changes. Exact P values are shown at the bottom of each table. Observations with P values equal to or less than 0.05 is considered significant and marked with an asterisk (*).

<u>**List of tables in this file**</u>

Table S1 A-D: Packed cell volume (PCV)
Table S2 A-D: Red blood cell (RBC) count
Table S3 A-D: Hemoglobin (Hb) concentration
Table S4 A-D Hematocrit values
Table S5 A-D: Low Plasma protein concentration
Table S6 A-D: High Plasma protein concentration
Table S7 A-D: Leukopenia
Table S8 A-D: Leukocytosis
Table S9 A-D: Lymphopenia
Table S10 A-D: Lymphocytosis
Table S11 A-D: Monocytosis
Table S12 A-D: Neutropenia
Table S13 A-D: Neutrophilia
Table S14 A-D: Eosinophilia
Table S15 A-D: Thrombocytopenia
Table S16: Reference values for the clinical pathology parameters

Table S1 A-D: Packed cell volume (PCV)

Table S1A

	**Low PCV**	**Normal PCV**	**Total**
**AP PCR +**	18	20	38
**AP PCR -**	26	73	99
**Total**	44	93	137

(P = 0.0244)*

Table S1B

	**Low PCV**	**Normal PCV**	**Total**
**AP POC+**	12	20	32
**AP POC -**	24	43	67
**Total**	36	63	99

(P= 1)

Table S1C

	**Low PCV**	**Normal PCV**	**Total**
**EC PCR +**	18	67	85
**EC PCR -**	26	26	52
**Total**	44	93	137

(P= 0.0007)*

Table S1 D

	**Low PCV**	**Normal PCV**	**Total**
**EC POC +**	30	35	65
**EC POC -**	6	28	34
**Total**	36	63	99

(P = 0.0077)*

Low PCV was significantly associated with AP PCR, EC PCR and EC POC positive test results

Table S2 A-D: Red blood cell (RBC) count

Table S2A

	Low RBC count	Normal RBC count	Total
**AP PCR +**	12	25	37
**AP PCR -**	28	81	109
**Total**	40	106	146

(P = 0.5225)

Table S2B

	Low RBC count	Normal	Total
**AP POC+**	9	22	31
**AP POC-**	15	53	68
**Total**	24	75	99

(P = 0.4592)

Table S2C

	Low RBC count	Normal	Total
**EC PCR +**	19	32	51
**EC PCR -**	11	74	85
**Total**	30	106	136

(P = 0.0013)*

Table S2D

	Low RBC count	Normal	Total
**EC SNAP+**	20	45	65
**EC SNAP -**	4	30	34
**Total**	24	75	99

(P = 0.0479)*

Low RBC count was significantly associated with EC PCR and EC POC positive test results

Table S3 A-D: Hemoglobin (Hb) concentration

Table S3A

	**Low Hb**	**Normal–High Hb**	**Total**
**AP PCR +**	18	20	38
**AP PCR -**	18	82	100
**Total**	36	102	138

(P =0.0009)*

Table S3B

	**Low Hb**	**Normal–high Hb**	**Total**
**AP POC+**	11	21	32
**AP POC-**	17	51	68
**Total**	28	72	100

(P =0.3484)

Table S3C

	**Low Hb**	**Normal-high Hb**	**Total**
**EC PCR +**	24	28	52
**EC PCR -**	12	76	88
**Total**	36	104	140

(P =0.0001)*

Table S3D

	**Low Hb**	**Normal-high Hb**	**Total**
**EC POC +**	24	42	66
**EC POC-**	4	30	34
**Total**	28	72	100

(P = 0.01)*

Low hemoglobin concentration is significantly associated with AP PCR, EC PCR and EC POC positive test results

Table S4 A-D Hematocrit values

Table S4A

	**Low HCT**	**Normal**	**Total**
**AP PCR +**	18	20	38
**AP PCR -**	20	75	95
**Total**	38	95	133

(P = 0.0051)*

Table S4B

	**Low HCT**	**Normal**	**Total**
**AP POC+**	11	20	31
**AP POC -**	19	47	66
**Total**	30	67	97

(P = 0.6380)

Table S4C

	**Low HCT**	**Normal**	**Total**
**EC PCR +**	26	25	51
**EC PCR -**	12	70	82
**Total**	38	95	133

(P = 0.0001)*

Table S4D

	**Low HCT**	**Normal**	**Total**
**EC POC +**	26	38	64
**EC POC -**	4	29	33
**Total**	30	67	97

(P = 0.0050)*

Low hematocrit values are significantly associated with AP PCR, EC PCR and EC POC positive test results

Table S5 A-D: Low Plasma protein concentration

Table S5A

	**Low plasma protein**	**Normal**	**Total**
**AP PCR +**	5	30	35
**AP PCR -**	10	51	61
**Total**	15	81	96

(P = 1)

Table S5B

	**Low plasma protein**	**Normal**	**Total**
**AP POC+**	12	19	31
**AP POC -**	8	40	48
**Total**	20	59	79

(P= 0.0359)*

Table S5C

	**Low plasma protein**	**Normal**	**Total**
**EC PCR +**	8	31	39
**EC PCR -**	7	50	57
**Total**	15	81	96

(P = 0.3913)

Table S5D

	**Low plasma protein**	**Normal**	**Total**
**EC POC +**	8	54	62
**EC POC -**	1	38	39
**Total**	9	92	101

(P = 0.1478)

Low Plasma protein concentration was significantly associated with AP POC positive test results.

Table S6 A-D: High Plasma protein concentration

Table S6A

	High Plasma protein concentration	Normal	Total
**AP PCR +**	3	30	33
**AP PCR -**	39	51	90
**Total**	42	81	123

(P = 0.0002)

Table S6B

	High Plasma protein concentration	Normal	Total
**AP POC+**	12	19	31
**AP POC -**	20	40	60
**Total**	32	59	91

(P = 0.6481)

Table S6C

	High Plasma protein concentration	Normal	Total
**EC PCR +**	13	31	44
**EC PCR -**	29	50	79
**Total**	42	81	123

(P = 0.5521)

Table S6D

	High Plasma protein concentration	Normal	Total
**EC POC+**	30	29	59
**EC POC -**	2	30	32
**Total**	32	59	91

(P = 0.0001)

High plasma protein concentration is significantly associated with AP PCR and EC POC positive test results

Table S7 A-D: Leukopenia

Table S7 A

	**Leukopenia**	**Normal**	**Total**
**AP PCR +**	3	31	34
**AP PCR -**	9	82	91
**Total**	12	113	125

(P = 1)

Table S7 B

	**Leukopenia**	**Normal**	**Total**
**AP POC+**	4	27	31
**AP POC -**	5	55	60
**Total**	9	82	91

(P = 0.4837)

Table S7 C

	**Leukopenia**	**Normal**	**Total**
**EC PCR +**	6	40	46
**EC PCR -**	6	73	79
**Total**	12	113	125

(P = 0.3554)

Table S7 D

	**Leukopenia**	**Normal**	**Total**
**EC POC+**	8	54	62
**EC POC-**	1	38	39
**Total**	9	92	101

(P= 0.1478)

Table S8 A-D: Leukocytosis

Table S8 A

	**Leukocytosis**	**Normal**	**Total**
**AP PCR +**	5	31	36
**AP PCR -**	3	84	87
**Total**	8	115	123

(P = 0.0466)*

Table S8 B

	**Leukocytosis**	**Normal**	**Total**
**AP POC+**	1	27	28
**AP POC -**	8	55	63
**Total**	9	82	91

(P = 0.2655)

Table S8 C

	**Leukocytosis**	**Normal**	**Total**
**EC PCR +**	6	40	46
**EC PCR -**	7	73	80
**Total**	13	113	126

(P = 0.5456)

Table S8 D

	**Leukocytosis**	**Normal**	**Total**
**EC POC+**	4	53	57
**EC POC -**	3	28	31
**Total**	7	81	88

(P = 0.6933)

Leukocytosis is significantly associated with AP PCR positive test results.

Table S9 A-D: Lymphopenia

Table S9 A

	Lymphopenia	Normal	Total
**AP PCR +**	2	31	33
**AP PCR -**	13	84	97
**Total**	15	115	130

(P = 0.3526)

Table S9 B

	Lymphopenia	Normal	Total
**AP POC+**	4	24	28
**AP POC -**	8	57	65
**Total**	12	81	93

(P = 0.7486)

Table S9 C

	Lymphopenia	Normal	Total
**EC PCR +**	6	38	44
**EC PCR -**	9	77	86
**Total**	15	115	130

(P = 0.5761)

Table S9 D

	Lymphopenia	Normal	Total
**EC POC +**	9	53	62
**EC POC -**	28	3	31
**Total**	37	56	93

(P = 0.0001)

Lymphopenia is significantly associated with EC POC positive test results.

Table S10 A-D: Lymphocytosis

Table S10 B

	Lymphocytosis	Normal	Total
**AP PCR +**	5	31	36
**AP PCR -**	3	84	87
**Total**	8	115	123

(P = 0.0466)*

Table S10 B

	Lymphocytosis	Normal	Total
**AP POC+**	4	24	28
**AP POC-**	3	57	60
**Total**	7	81	88

(P=0.2024)

Table S10 C

	Lymphocytosis	Normal	Total
**EC PCR +**	8	38	46
**EC PCR -**	0	77	77
**Total**	8	115	123

(P = 0.0003)*

Table S10 D

	Lymphocytosis	Normal	Total
**EC POC +**	4	53	57
**EC POC -**	3	28	31
**Total**	7	81	88

(P = 0.6933).

Lymphocytosis is significantly associated with AP PCR and EC PCR positive test results

Table S11 A-D: Monocytosis

Table S11 A

	Monocytosis	Normal	Total
**AP PCR +**	2	31	33
**AP PCR -**	2	85	87
**Total**	4	116	120

(P = 0.3032)

Table S11 B

	Monocytosis	Normal	Total
**AP POC+**	0	26	26
**AP POC -**	2	60	62
**Total**	2	86	88

(P = 1)

Table S11 C

	Monocytosis	Normal	Total
**EC PCR +**	4	42	46
**EC PCR -**	0	74	74
**Total**	4	116	120

(P = 0.0199)*

Table S11 D

	Monocytosis	Normal	Total
**EC POC+**	2	54	56
**EC POC -**	0	32	32
**Total**	2	86	88

(P= 0.5319)

Monocytosis is significantly associated with EC PCR positive test results.

Table S12 A-D: Neutropenia

Table S12 A

	**Neutropenia**	**Normal**	**Total**
**AP PCR +**	1	32	33
**AP PCR -**	3	89	92
**Total**	4	121	125

(P= 1)

Table S12 B

	**Neutropenia**	**Normal**	**Total**
**AP POC+**	0	31	31
**AP POC -**	2	58	60
**Total**	2	89	91

(P=0.5458)

Table S12 C

	**Neutropenia**	**Normal**	**Total**
**EC PCR +**	1	45	46
**EC PCR -**	3	76	79

(P = 1)

Table S12 D

	**Neutropenia**	**Normal**	**Total**
**EC POC +**	1	61	62
**EC POC -**	1	28	29
**Total**	2	89	91

(P = 0.5382)

Table S13 A-D: Neutrophilia

Table S13 A

	Neutrophilia	Normal	Total
**AP PCR +**	5	32	37
**AP PCR -**	8	89	97
**Total**	13	121	134

(P = 0.3466)

Table S13 B

	Neutrophilia	Normal	Total
**AP POC+**	1	31	32
**AP POC-**	1	58	59
**Total**	2	89	91

(P= 1)

Table S13 C

	Neutrophilia	Normal	Total
**EC PCR +**	6	45	51
**EC PCR -**	7	76	83
**Total**	13	121	134

(P = 0.5579)

Table S13 D

	Neutrophilia	Normal	Total
**EC POC+**	4	61	65
**EC POC -**	5	28	33
**Total**	9	89	98

(P = 0.1594)

Table S14 A-D: Eosinophilia

Table S14 A

	Eosinophilia	Normal	Total
**AP PCR +**	14	24	38
**AP PCR -**	15	85	100
**Total**	29	109	138

(P = 0.0090)*

Table S14 B

	Eosinophilia	Normal	Total
**AP POC+**	11	21	32
**AP POC-**	11	57	68
**Total**	22	78	100

(P = 0.0679)

Table S14 C

	Eosinophilia	Normal	Total
**EC PCR +**	16	36	52
**EC PCR -**	13	73	86
**Total**	29	109	138

(P = 0.0332)*

Table S14 D

	Eosinophilia	Normal	Total
**EC POC+**	15	51	66
**EC POC -**	7	27	34
**Total**	22	78	100

(P = 1)

Eosinophilia is significantly associated with AP PCR and EC PCR positive test results.

Table S15 A-D: Thrombocytopenia

Table S15 A

	**Thrombocytopenia**	**Normal**	**Total**
**AP PCR +**	26	11	37
**AP PCR -**	46	53	99
**Total**	72	64	136

(P = 0.0199)*

Table S15 B

	**Thrombocytopenia**	**Normal**	**Total**
**AP POC+**	28	4	32
**AP POC -**	28	38	66
**Total**	56	42	98

(P = 0.0001)*

Table S15 C

	**Thrombocytopenia**	**Normal**	**Total**
**EC PCR +**	44	8	52
**EC PCR -**	28	56	84
**Total**	72	64	136

(P = 0.001)*

Table S15 D

	**Thrombocytopenia**	**Normal**	**Total**
**EC POC +**	44	22	66
**EC POC -**	12	20	32
**Total**	56	42	98

(***P = 0.0088)****

Thrombocytopenia is significantly associated with AP PCR, EC PCR, AP POC and EC POC positive test results.

Table S16: Reference values for the clinical pathology parameters

Parameter	Reference values
Packed cell volume (PCV)	37-55 (%)
Red Blood cell count (RBC)	5.5-8.5 ($x10^{12}$/L)
Hemoglobin (HGB)	12-18 (g/dL)
Hematocrit (HCT)	37-55(%)
Mean corpuscular volume (MCV)	60-77 (fL)
Mean Corpuscular hemoglobin concentration (MCHC)	31-39 (g/dL)
Total protein (TP)	6-7.5 (g/dL)
White blood cell count (WBC)	6-17 ($x10^9$/L)
Lymphocytes	1-4.8($x10^9$/L)
Monocytes	0.20-1.5 ($x10^9$/L)
Neutrophils	3-12 ($x10^9$/L)
Eosinophils	0-0.8 ($x10^9$/L)
Basophils	0.0.4 ($x10^9$/L)
